# Supplementary material for: Hemolysis induced by Left Ventricular Assist Device is associated with proximal tubulopathy
Source: PLoS One. 2020 Nov 30;15(11):e0242931. doi: 10.1371/journal.pone.0242931 (PMC7703997; doi:10.1371/journal.pone.0242931)
Supplement: S1 Table — Values are medians [25th-75th percentile]. ECMO: Extracorporeal Membrane Oxygenation. LDH: Lactate Dehydrogenase. AKI: Acute Kidney Injury. (DOCX) [file pone.0242931.s001.docx]

| Characteristics | Hemodialysis after LVADs implantation  N=7 | No need for hemodialysis  N=26 | p |
| --- | --- | --- | --- |
| Age (year) | 65.0 [59.0 – 71.0] | 60.0 [56.5 – 67.5] | 0.35 |
| Baseline creatinine, µmol/L | 73 [60 – 127] | 112 [81 – 152] | 0.11 |
| Baseline proteinuria, g/g | 0.6 | 0.6 |  |
| Indication, n (%)   - Bridge to transplantation - Destination | 4 (57.1)  3 (42.9) | 21 (80.8)  5 (19.2) | 0.32 |
| Cardiopathy, n (%)   - Ischemic - Dilative - Myocarditis | 4 (57.1)  3 (42.9)  0 | 17 (65.4)  8 (30.8)  1 (3.8) | 0.59 |
| INTERMACS classification, n (%)   - 1 - 2 - 3 - 4 - 5-7 | 1 (14.3)  3 (42.9)  3 (42.9)  0  0 | 6 (23.1)  12 (46.2)  3 (11.5)  4 (15.4)  1 (3.8) | 0.34 |
| Temporary mechanical circulatory support, n (%)   - ECMO - Impella - None | 4 (57.1)  2 (26.6)  1 (14.3) | 14 (53.8)  8 (30.8)  4 (15.4) | 0.99 |
| AKI (n of episode/year) | 4.6 [2.8 – 5.3] | 2.1 [0.4 – 3.7] | 0.06 |
| Plasma creatinine at the end of follow-up, µmol/L | 125 [60 – 150] | 101 [87 – 150] | 0.66 |
| LDH, IU/L | 361 [335 – 709] | 385 [334 – 689] | 0.94 |
| Proximal tubulopathy, n (%)   - Yes - No | 3 (42.9)  4 (57.1) | 3 (11.5)  23 (88.5) | 0.09 |

S1 Table. characteristics of patients according to the need of acute hemodialysis after LVADs implantation. Values are medians [25th-75th percentile]. ECMO: Extracorporeal Membrane Oxygenation. LDH: Lactate Dehydrogenase. AKI: Acute Kidney Injury.
